# Diagnosis and Management of Neonatal Bacterial Sepsis: Current Challenges and Future Perspectives

**DOI:** 10.3390/tropicalmed9090199

**Published:** 2024-08-28

**Authors:** Domenico Umberto De Rose, Maria Paola Ronchetti, Ludovica Martini, Jole Rechichi, Marco Iannetta, Andrea Dotta, Cinzia Auriti

**Affiliations:** 1 Neonatal Intensive Care Unit, “Bambino Gesù” Children’s Hospital IRCCS, 00165 Rome, Italy; domenico.derose@opbg.net (D.U.D.R.); mariapaola.ronchetti@opbg.net (M.P.R.); ludovica.martini@opbg.net (L.M.); andrea.dotta@opbg.net (A.D.); 2 PhD Course in Microbiology, Immunology, Infectious Diseases, and Transplants (MIMIT), Faculty of Medicine and Surgery, “Tor Vergata” University of Rome, 00133 Rome, Italy; 3 Neonatal Sub-Intensive Care Unit, “Bambino Gesù” Children’s Hospital IRCCS, 00165 Rome, Italy; jole.rechichi@opbg.net; 4 Infectious Disease Clinic, Policlinico “Tor Vergata” University Hospital, 00133 Rome, Italy; marco.iannetta@uniroma2.it; 5 Department of System Medicine, “Tor Vergata” University of Rome, 00133 Rome, Italy; 6 Pediatrics Department, Saint Camillus International University of Health Sciences, 00131 Rome, Italy; 7 Casa di Cura Villa Margherita, 00161 Rome, Italy

**Keywords:** early-onset sepsis, late-onset sepsis, sepsis calculator, blood culture, PCR assays, biomarkers, c-reactive protein, procalcitonin, presepsin, antibiotic lock therapy

## Abstract

Sepsis remains the second cause of death among neonates after the pathological consequences of extreme prematurity. In this review we summarized knowledge about pathogens causing early-onset sepsis (EOS) and late-onset sepsis (LOS), the role of perinatal risk factors in determining the EOS risk, and the tools used to reduce unnecessary antibiotics. New molecular assays could improve the accuracy of standard blood cultures, providing the opportunity for a quick and sensitive tool. Different sepsis criteria and biomarkers are available to date, but further research is needed to guide the use of antibiotics according to these tools. Beyond the historical antibiotic regimens in EOS and LOS episodes, antibiotics should be based on the local flora and promptly modulated if specific pathogens are identified. The possibility of an antibiotic lock therapy for central venous catheters should be further investigated. In the near future, artificial intelligence could help us to personalize treatments and reduce the increasing trend of multidrug-resistant bacteria.

## 1. Background: Why Talk about Neonatal Sepsis?

Bacterial sepsis still represents one of the main causes of death, disability, and consumption of healthcare resources for millions of people every year in each age category [1].

In the pediatric and neonatal age, despite a global reduction in overall mortality in the last twenty years, the largest reports indicate a growing prevalence of severe sepsis [2], which also reflects a growth in the population of individuals with chronic co-morbidities, and therefore more vulnerabilities, and there is a growing trend of germs’ resistance to antibiotics and an increase in opportunistic infections. For biological reasons related to the functional maturity of the immune system, the most fragile people, such as newborns, are the most affected by sepsis, even in high-income countries. The risk of acquiring sepsis is inversely proportional to gestational age (GA), with the most premature newborns being at the highest risk of it [3]. The other important risk factor of sepsis is the pre-term newborns’ need for invasive procedures to survive during hospitalization in Neonatal Intensive Care Units (NICUs). In the context of intensive care, it is frequently necessary to interrupt natural barriers against infection: the skin is affected by plasters, catheters, surgery, etc., and the mucosa through ventilation, nutrition, and drugs. This naturally compromises the child’s already weak possibilities of defense against bacterial invasion. Within the neonatal population, very premature babies, with a gestational age of 23–27 weeks, survive longer now than in the past [4].

Nonetheless, sepsis remains the second cause of death among those babies after the pathological consequences of extreme prematurity. It is estimated that 12–13% of deaths among neonates of 22–40 weeks are attributable to this devastating pathology, and approximately 40% of all deaths involve neonates born before 25 weeks [5].

## 2. Where Does the Current Classification of Neonatal Sepsis Come from?

Neonatal sepsis is defined in a variety of ways, including microbiological culture, laboratory testing, and clinical symptoms, as opposed to adult and pediatric sepsis, which is defined based on organ failure [6]. Neonatologists divide neonatal sepsis by a temporal criterion of the onset of symptoms. Neonatal sepsis is defined as early (Early Onset Sepsis—EOS) if the clinical symptoms appear in the first 72 h of life, and late (Late Onset Sepsis—LOS) if they arise after the first 72 h of life, or in any case after 48 h from admission to the ward. This subdivision is useful to crudely but quickly understand the origin of the infection (maternal or environmental), the category of responsible germs, and to start an empirical but roughly oriented antibiotic therapy. In fact, the germs responsible for EOS sepsis are mostly different from those responsible for LOS sepsis [7,8].

The timely start of antibiotics, in terms of hours, is one of the ways to reduce mortality from sepsis in the newborn, as well as in the older child, and it is fundamental to the early reduction of infection, as is the choice of antibiotics with a targeted antibacterial spectrum to the germ, once the results of the microbiological tests are available [9].

### 2.1. Early-Onset Sepsis

EOS is mostly caused by maternal–fetal transmission of the germ, a mechanism of infection also called vertical transmission. The onset of symptoms is usually within 72 h from birth, but of the seven randomized controlled trials (RCTs) that defined EOS, one study stated early onset as arising <48 h of life, five studies as <72 h, and one study as <5 days of life. Conversely, of the eight available RCTs that outlined LOS, all identified late-onset sepsis episodes as arising ≥72 h of life [6].

Group B Streptococcus (GBS) and *Escherichia coli* represent the most common causing agents of EOS, accounting for 38% and 23% of episodes, respectively [10]. These statistics are reversed in the pre-term population, as *E. coli* accounts for 50% of cases, while GBS is responsible for only 20–25% of cases [11]. Although GBS is more common overall, *E. coli* is still the primary cause of EOS-related morbidity and death among pre-term infants [12,13].

Since 1990, the year in which screening of pregnant women colonized by the bacterium began, and the use of intrapartum antibiotic prophylaxis was introduced, there has been a notable reduction in infections due to GBS. GBS remains, in any case, a major cause of EOS and neonatal and childhood meningitis worldwide. Among the ten known GBS serotypes (Ia, Ib, and II-IX), serotypes Ia, III, and V are the most widespread in newborns and infants up to 90 days of life. Up to 50% of newborns who contract GBS meningitis and survive it have outcomes of variable severity. To date, several preventive strategies have been adopted to reduce early-onset GBS disease (EOD, with the onset at 0–6 days of life), and between 1990 and 2015 in the United States, the incidence of GBS sepsis decreased from 1.8 to 0.23 per 1000 live births [14]. In the United States, pre-natal vaginal–rectal screening at 35 and 37 weeks of gestation, and the administration of intrapartum antibiotic prophylaxis (IAP), have been very effective at reducing the incidence of EOD. This drastic reduction is also observed in other high-income countries that adopt pre-natal screening, such as Italy, for which we have data from the regional surveillance system of Emilia Romagna. In Italy, the estimated incidence of streptococcal EOS is 0.20/1000 live births [15]. However, the reduction was achieved through the extensive use of intrapartum antibiotics (approximately 35% of pregnant women in Emilia-Romagna receive an IAP). Other Northern European countries (such as England and Holland), or African countries (South Africa), do not screen women before delivery, and administer intrapartum antibiotic prophylaxis only to those with risk factors [16]. With this second strategy, the EOD did not reduce, and in some cases it even increased. Finally, no strategy had any effect on late-onset GBS infection (LOD, onset at 7–90 days of life), the incidence of which remains unchanged throughout the world. Late-onset infection presents with meningitis (with frequent long-term outcomes) in almost half of cases. It is, therefore, clear that strategies aimed at reducing the use of intrapartum antibiotics and preventing both types of GBS infection (EOD and LOD) are necessary. Currently, research is focused on the preparation of vaccines, but to date, no type of vaccine has yet been marketed [17]. It is estimated that to detect a 75% reduction in EOD and LOD in countries with a disease incidence of over 1/1000 births, it would be necessary to enrol approximately 60,000 pregnant women to study the effectiveness of the vaccine if this protects against 90% of circulating serotypes. This measure considers the difficulty of studying the effectiveness of vaccines against GBS, which nevertheless reached phase II [17].

### 2.2. Late-Onset Sepsis

**Figure 1:**
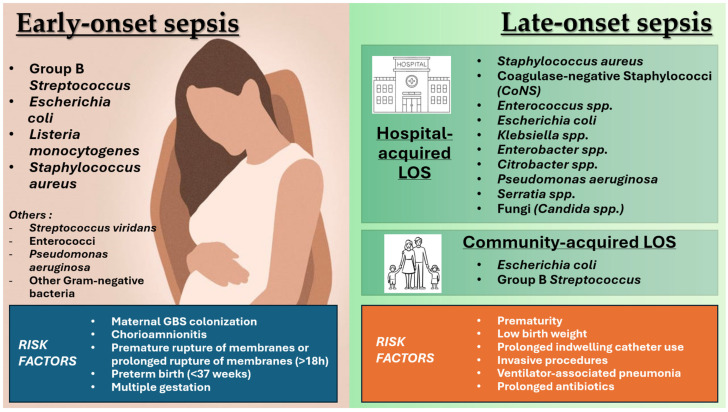
Main pathogens involved in early-onset and late-onset sepsis.

LOS occurs after 72 h of life, during hospitalization, and mainly affects newborns at particular risk, such as pre-term newborns hospitalized in the NICU. LOS incidence varies from 15% to 36% of hospitalized newborns, depending on the care context. In the genesis of these infections, biological factors such as prematurity and all invasive procedures play the primary role, and the mortality rate in infected patients is approximately four times that of non-infected patients. The responsible germs of LOS are different from those of EOS. Usually, hospital-acquired LOS are typically caused by pathogens from intensive care flora or saprophytic of the skin, such as Gram-positive bacteria (69%), Coagulase negative Staphylococci (CoNS) (40%), Gram-negative bacteria (29%), or fungi (3%). Conversely, in a recent population-based study by Giannoni et al., *E. coli* and GBS accounted for 41% and 41% of community-acquired LOS episodes, defined as LOS with the onset ≤48 h after admission to the ward. (Figure 1) [10].

However, the paradigm EOS/LOS is based on observed epidemiological data from high-income countries (HIC), whereas this concept may be less useful for predicting the pathogen spectrum of neonatal sepsis in low-income and middle-income countries (LIC and MIC, respectively) [18]. Although worldwide incidence data are lacking, the overall incidence of newborn sepsis is lower in HIC than in LIC [18]. Pathogens such as Klebsiella spp. may account for a significant ‘excess’ burden of newborn sepsis in LIC settings, outweighing organisms more often seen in HIC settings.

Currently, the irrational use of antibiotics, often used for “preventive purposes”, is leading to the emergence of antibiotic-resistant bacterial strains, which themselves cause intractable sepsis.

Intrapartum antibiotic prophylaxis for GBS status may change the etiology of EOS, with some studies showing *E. coli* surpassing GBS as a primary cause of EOS [19]. Administered antibiotics impact the newborn’s microbiome, tipping the balance toward horizontally rather than vertically acquired microbes [20]. For these reasons, there is a great deal of research on the reduction of the use of prophylactic antibiotics, the dissemination of the principles of antibiotic stewardship, and the identification of the criteria for starting antibiotic therapy and the newborns who require it.

Neonatologists are, therefore, invested with a whole series of great responsibilities, from raising awareness among the population around the opportunity to adhere to screening programs, to therapeutic decisions regarding the start and stop of antibiotic therapies in hospitalized newborns, to the drafting of guidelines for the prevention of neonatal sepsis. What we wanted to underline is that, beyond the definitions of EOS and LOS which can be useful for a first orientation, what is important is to identify appropriate criteria for the use of antibiotics in both situations.

## 3. Neonatal Sepsis: Some Numbers

We are in the historical time of the safety of healthcare treatments and the appropriateness of interventions. In this context, “Get zero” for infections and mortality related to infections is a primary objective, and infections leave a large space for intervention. In fact, between 20% and 30% of healthcare-associated infections would be preventable. Therefore, there is still a lot to do in neonatology.

The global pooled incidence of neonatal sepsis in the world is 22/1000 live births (1–4/1000 live births in high-income countries, and 49–170 cases per 1000 births in low-income countries), with a death rate of 11 to 19 percent [1].

Neonatal sepsis rates increase as gestational age (GA) decreases, with a higher incidence of culture-proven episodes among very low birth weight (VLBW) pre-term infants. In VLBW infants, an incidence of EOS has been reported in 0.5–3.1% of neonates, while LOS episodes occurred in 2–32% [19,21,22].

Sepsis is an important cause of death, with mortality rates varying on average from 11% to 19% in infected newborns. It can reach 35% in cases of EOS [23], and varies from 18 to 36% in cases of LOS [24].

Both EOS and LOS episodes have serious long-term implications for newborns, and the probability of long-term adverse outcomes increases when the gestational age decreases, being higher in pre-term infants than in more mature babies. A large two-year study by Stoll et al. examined the current epidemiology of EOS across the GA spectrum. The aim was to evaluate the effectiveness of obstetric prevention measures, and clinical assessment and treatment on neonatal survival outcomes. Two hundred and thirty-five EOS cases were identified in a cohort of 217,480 infants born in academic centers in 14 states, with an estimated EOS incidence of 1.08/1000 live births. Among 235 cases of infections, 131 (55.7%) were observed among babies younger than 37 weeks of GA. Thirty-eight of the 131 infected infants with GA of less than 37 weeks (29.0%) died, while no infant born at full term died [19]. Gonçalves et al., in a very large meta-analysis, estimated the impact of maternal GBS colonization, cases of invasive GBS disease, deaths in infants under 3 months of age, children surviving invasive GBS disease with neurodevelopmental impairment, and maternal invasive GBS cases in 183 countries in 2020. The authors reported that, out of an estimated 19,700 million women colonized by GBS, there were 231,800 cases of early-onset and 162,200 cases of late-onset GBS invasive disease in neonates and infants, with mortality ranging from 58,300 to 91,900 deaths in relation to the presence of a skilled attendant at delivery. Of the newborns who survived the infection, 37% showed neuro-developmental impairment of varying severity [25].

Alshaikh et al., in a systematic review and meta-analysis of 17 studies on neurological damage associated with very low birth weight (VLBW), reported that newborns weighing <1500 g with neonatal sepsis had an increased risk of one or more serious disabilities, including cerebral palsy [3]. In total, neonatal sepsis causes disability in 39% of those affected [26].

## 4. The Role of Perinatal Risk Factors in Determining the Risk of Early-Onset Sepsis

Perinatal risk factors (RFs) are historically considered an adequate reason for evaluating an infant for EOS, with perinatal asphyxia or intrauterine distress, meconium contamination in amniotic fluid, GBS colonization in pregnant women, chorioamnionitis, premature rupture of membranes, lower gestational age, maternal urinary tract or reproductive tract infection, perinatal fever, very low birth weight, and vaginal examinations ≥ 3 times potentially increasing the risk of EOS [27].

Indeed, up to 15% of newborns are tested, and around half of them receive empiric antibiotics while ruling out EOS [28].

Serial clinical observation (SCO) during the first 48 h of life has recently gained consensus among clinicians, with laboratory evaluation or empirical antibiotic therapy only if clinical signs of illness develop [29].

Selecting newborns only based on maternal RFs may result in missing cases of EOS. A large, multicenter investigation in Italy reported 48 occurrences of confirmed GBS EOS among 265,508 live births handled using a SCO strategy in the nursery; 15 out of 48 patients (31.2%) had no RFs for EOS and experienced EOS symptoms (three had severe distress) while in the hospital. Most of them (12/15, 80%) appeared normal at birth but later acquired symptoms [15].

## 5. Do the Symptoms of Neonatal Sepsis Vary with the Type of Early or Late Infection?

Whatever the type of neonatal sepsis, the symptoms at onset are generally not specific to the type of infection. We usually observe an increased oxygen requirement due to worsening respiratory distress, apnea crisis, hyporeactivity up to the lethargic state, feeding difficulties, temperature instability, prolonged capillary refill time, hyperglycemia or hypoglycemia, lactic acidosis, cyanosis, and shock. In more fragile patients, such as those with a very low birth weight or severely pre-term infants, progression from mild symptoms to death may occur within less than 24 h, especially for infections with gram-negative bacteria such as *E. coli* and *Klebsiella*. The clinical onset may also be characterized by paralytic ileus, with a reduction of intestinal peristalsis.

When neurological involvement is present, dystonia, convulsive seizures, high-pitched crying, and tense fontanelles may occur. Meningitis is often associated with bacteremia in EOS early-onset sepsis, but in a third of cases it may occur later without associated bacteremia. Given that, a lumbar puncture should be performed in case of suspected EOS, especially in pre-term newborns, but neonatologists often avoid this test, which, even in the largest US series, is carried out in just over 66% of newborns [19]. Concerning LOS, lumbar puncture should always be performed in case of symptoms suspected of involving the central nervous system [30]. Moreover, if LOS is suspected, the limbs and joints should be examined for signs of osteomyelitis and septic arthritis, sometimes related to the positioning of intravascular accesses or other devices.

Urinary tract infection is a common cause of infection in children with sepsis. Hence, urine cultures should be acquired in all patients before antibiotic therapy.

## 6. Neonatal Early-Onset Sepsis Calculator

The neonatal EOS-risk sepsis calculator (NSC), developed by Kaiser Permanente (San Leandro, CA, USA), has been the most discussed strategy in recent years for lowering antibiotic treatment for suspected EOS in late pre-term and term newborns (≥34 weeks gestational age) [31].

The interactive NSC (available at: https://neonatalsepsiscalculator.kaiserpermanente.org/, accessed on 20 July 2024) calculates the likelihood of early-onset sepsis per 1000 newborns by entering values for (1) EOS incidence in the considered population, (2) selected maternal risk factors (gestational age, highest maternal antepartum temperature, hours of rupture of membranes, maternal status for GBS, type of received intrapartum antibiotics), and (3) the infant’s clinical presentation in the first 12 h of life (well-appearing, equivocal, clinical illness).

Its introduction into clinical practice has been demonstrated to lower antibiotic prescriptions objectively [4], despite inadequate safety evidence [32]. Indeed, some EOS cases can be missed, mostly when information about membrane rupture is not accurate, also, this scoring system is limited by the gestational age and is applicable only in EOS. Concurrently, the NSC seems to recommend more antibiotics than the SCO approach without improving neonatal outcomes [33].

Studies evaluating a sepsis calculator-guided approach to discontinuing antibiotics are still lacking.

## 7. Clinical Scoring Tools

By combining various combinations of inflammatory response indicators, laboratory evaluations, and physical examination results, researchers have sought to establish and verify so-called “sepsis scores”, nevertheless, a single score has not shown to be consistently accurate [34].

Sepsis was defined as a “life-threatening organ dysfunction caused by a dysregulated host response to infection” in the Third International Consensus Definitions for Sepsis and Septic Shock (Sepsis-3) published in 2015. It was linked to Systemic Inflammatory Response Syndrome (SIRS) and progressed toward multiple organ failure [35]. The task group concentrated on adult patients to provide revised criteria that would be comparable to pediatric populations in a later phase. When Hofer et al. previously assessed if the sepsis and SIRS criteria applied to infected full-term babies, they discovered that only 53 percent of the septic patients with positive cultures met the common definition [36]. In fact, some characteristics that are present in a newborn’s physiology from birth are included in the definition of SIRS, including tachycardia, tachypnoea, a rise in bilirubin exceeding 5 mg/dL, and oliguria. The Sequential [Sepsis-related] Organ Failure Assessment score (SOFA score), which is not appropriate for infants for the reasons previously indicated, was used to characterize the evolutionary aspects of the SIRS. Moreover, neonatologists with expertise in caring for babies were not included among the specialists gathered, and newborns were defined as those under the age of 18. Pre-term neonates were also not included [35].

As a primary cause of morbidity and death, organ dysfunction (host response) is the focal point of the current definition of sepsis. When a newborn needs intensive care from delivery, it might be difficult to diagnose organ malfunction since there is no baseline data to gauge postpartum change from. Furthermore, pre-term babies may be unstable from the moment of delivery, infection or no infection. The best way to test for organ malfunction, particularly in pre-term babies, is unclear. We should consider the fact that the rate of positive blood cultures during sepsis in infants is around 9% if positive blood cultures are the gold standard for diagnosis [37].

Different sepsis criteria are available in the literature (Table 1), such as:

- Tollner sepsis score;
- Hematologic Scoring System;
- International Pediatric Consensus Conference statement on sepsis and organ dysfunction in pediatrics;
- NNF clinical practice guidelines (National Neonatology Forum, India);
- NEO–KISS Sepsis score;
- Neonatal Sequential Organ Failure Assessment (nSOFA) score, designed by Wynn and Polin to predict mortality from LOS in pre-term, very-low-birth-weight (VLBW) infants [38];
- NeoSep Severity Score, from the recent worldwide observational NeoOBS study, which included data from 3204 babies in low- and middle-income nations, emphasizing the critical need for clinical trials to guide the worldwide use of antibiotics for neonatal sepsis [39].

In particular, the nSOFA score has become the most popular recently. It uses three categorical criteria to specifically and objectively describe dynamic changes in (1) the need for mechanical ventilation and oxygen, (2) the need for inotropic support (for presumed adrenal insufficiency or catecholamine-resistant shock), and (3) the presence and degree of thrombocytopenia. A score higher than 4 in LOS was associated with higher mortality [38], as confirmed by Fleiss et al. [40] and Poggi et al. [41]. In particular, the likelihood ratio for mortality progressively increased as the nSOFA score increased (2-fold with a nSOFA score ≥ 2, 4-fold with a score ≥ 6, 8-fold with a score ≥ 8, and 16-fold with a score ≥ 10) [40]. However, we believe that since numerous signs of respiratory or cardiovascular instability are already present at delivery in pre-term newborns, regardless of infection, it may not have the same accuracy for early-onset cases as it does for late-onset cases.

Interestingly, the authors from the NeoOBS study distinguished (1) the NeoSep Severity Score, as a baseline for predicting the 28-day mortality based on characteristics identified at the time of sepsis presentation, and (2) the NeoSep Recovery Score to forecast the daily risk of death after intravenous antibiotic treatment based on daily updated assessments of clinical condition [39].

## 8. Blood Cultures and New Molecular Methods

Blood culture (BC) is considered the gold standard for diagnosing sepsis. Blood volume is considered the major factor influencing the possibility of having a positive BC, and available recommendations suggest a wide range for optimal blood volume. For neonates, a blood volume of 0.5–1 mL is usually suggested because 0.5 mL is the minimal volume validated by some companies [42,43]. However, inadequate blood volume is linked to a higher rate of false-negative BC and a greater proportion of contaminants that are identified [42].

“Culture-negative sepsis” is a term used to characterize newborns with sterile blood cultures but a clinical course suggestive of sepsis. These newborns are frequently diagnosed with sepsis and given a complete course of antibiotics. However, this causes overdiagnosis of newborn sepsis and misuse of antibiotics, especially in pre-term neonates and when some biomarkers are increased [44,45].

Negative cultures are a challenge for clinicians, who must distinguish between true sepsis and sepsis-like diseases (non-infectious or viral) that may not require antibiotics. On the other hand, antibiotics are required for focal infections with negative blood cultures, such as meningitis, urinary tract infections, pneumonia, peritonitis, and septic arthritis [37].

Two blood cultures taken simultaneously from two different sites improve the pathogen detection rate compared to the routine practice of single BC, but sometimes obtaining BC from a single site is already challenging in critically ill neonates [46]. Furthermore, while dual-site culture practices may be useful, clinicians should balance the gain in sensitivity of bacteremia detection against additive contamination risk [47].

In most NICUs, only one aerobic BC bottle is usually processed, whereas few sites routinely also use anaerobic BC bottles [48]. Based on the results of a recent study on 3665 infants, including anaerobic culture bottles could lead to the identification of pathogens not recovered in the aerobic bottle, as well as the earlier identification of pathogens [49].

Most pathogenic BCs obtained before starting antibiotic treatments from term and late pre-term infants returned a positive result within 24–36 h of incubation, based on results available in the literature: the Time to Positivity (TTP) could inform decisions on antibiotic administration and help in antibiotic stewardship, and empirical antibiotic treatment could already be withdrawn 24 h after obtaining blood cultures [50,51,52,53,54].

New Real-Time Polymerase Chain Reaction (PCR) assays have the potential to be a valuable additional tool for the diagnosis of neonatal sepsis [55].

The FilmArray Blood Culture Identification (BCID) panel (bioMérieux, Marcy l’Etoile, France) is a multiplex PCR test with a 2-min hands-on time and a 1-h turnaround time that enables detection of bloodstream infection (BSI). The BioFire FilmArray BCID2 panel has 43 molecular targets for BSI, including 15 Gram-negative bacteria, 11 Gram-positive bacteria, seven yeast species, and 10 antimicrobial resistance genes. The BCID2 Panel shows good diagnosis accuracy when compared to conventional microbiological approaches [56,57,58,59].

If blood cultures do not yield positive findings, PCR assays can identify pathogens within two hours, with the target of a single organism (e.g., *S. aureus*) or broad groups of pathogens. For example, the T2 Magnetic Resonance Technology (T2MR) is a direct molecular assay for the identification of BSI pathogens, which can help to overcome the limits of blood culture (BC), such as diagnostic accuracy, blood volumes required, and turnaround time. Including these new assays with BC in the diagnostic pathway should assist in overcoming present diagnostic problems, even in newborns whose clinical vulnerability necessitates a quick and sensitive approach [60].

Collaboration with the microbiology team is crucial to reducing the spectrum of antimicrobial therapy and preventing prolonged anti-Gram-positive medications in the case of Gram-negative bacteria, or vice versa, minimizing the development of antibiotic resistance [45].

## 9. The Use of Sepsis Biomarkers

Because of the significance of sepsis on neonatal mortality, measures to prevent its start and progression are vital, and early, targeted therapy has emerged as a crucial component of severe sepsis treatment bundles. A delay in starting antibiotic treatment is an independent risk factor for mortality and cardiovascular dysfunction in neonates, but this should not be translated into a very low treatment threshold and overtreatment [61].

According to the recent multicenter NO-MAS-R research, 80% of neonates receiving antibiotic treatment on the day of observation had been on therapy for more than 72 h, regardless of the findings of the cultures, with a median duration of 7 days [62].

However, early indications of sepsis in a newborn are frequently mild and non-specific, but the clinical course is quick and severe [63]. When sepsis is suspected, and general conditions are not reassuring, antibiotics should be promptly started, but afterward, the antibiotic treatment is to be discussed again if no more is indicated [45].

The availability of sepsis biomarkers that can alert physicians to early identification of neonatal sepsis could enhance the short and long-term outcomes of real sepsis patients, while also reducing the indiscriminate and harmful use of preventative antibiotics [64].

An ideal biomarker should have high sensitivity, specificity, and negative predictive values. Moreover, it would be fantastic if this biomarker could be dosed bedside, on a small blood volume, increasing the chances of using it even in low-birth-weight infants. No one biomarker has been found that fits the majority of these characteristics, and this futuristic tool appears not to exist or has yet to be discovered [65].

C-reactive protein (CRP) and procalcitonin (PCT) are the most widely used biomarkers of neonatal sepsis, but their accuracy is still disputed [66]. In recent years, many NICUs have focused their interest on P-SEP due to the ability to measure it with a point-of-care device and the apparent absence of confounding variables that alter its levels, although further studies are still needed [67,68]. The dilemma is whether these inflammatory markers are game-changers or merely gimmicks in LOS episodes, as seems in the case of early-onset sepsis, and antibiotic treatment should not be prolonged according to their values [69].

### 9.1. Biomarkers in Early-Onset Sepsis

Among different biomarkers studied in early-onset sepsis (EOS) episodes, white blood cell count (WBCC) and the ratio between immature and total neutrophils, were among the earliest studied markers. Neutrophilic leukocytosis is generally considered normal at birth, and there has been a reported poor sensitivity of 39–49%, combined with a moderate to good specificity of 73–81%, in detecting EOS episodes [70].

CRP is an acute-phase reactant produced by the liver in reaction to inflammatory cytokines (mainly interleukin 6) produced by white blood cells responding to microbial pyrogens, with most laboratories using a cut-off of 5 to 10 mg/L [71]. CRP levels rise 10 to 12 h after pathogen contact, peaking in 48 to 72 h [11]. CRP levels recover to normal within 3–7 days, although they can be influenced by non-infectious inflammatory triggers (such as perinatal hypoxia) and delivery [72]. Indeed, CRP levels can be similar in infants with positive and negative blood cultures [52]. CRP has been widely studied in EOS, with high heterogeneity between studies, threshold effects, and poor pooled sensitivity (58%), especially within the first 12 h. The most promising results were obtained for CRP samples between 12 and 24 h after birth, with sensitivity of 76–89% and specificity of 75–87% among the different subgroup analyses [70].

PCT is a precursor of the hormone calcitonin: in normal conditions, the PCT gene (CALC-1) is almost exclusively expressed by neuroendocrine thyroid C cells, and produced PCT is stored in the Golgi apparatus, which explains the low levels seen in circulation. CALC-1 is up-regulated, and hence expressed in all organism cells during systemic infections, releasing higher quantities of PCT into circulation [73]. PCT was sensitive enough to detect sepsis episodes significantly sooner than CRP [74], as it is detectable 3 h after exposure and peaks at 6 h [11]. After around 12 h, a plateau occurs, and PCT levels revert to normal within 2–3 h. However, interpreting procalcitonin levels in neonates is made harder by a physiological increase in the first 48 h of life, in addition to other perinatal variables (such as chorioamnionitis, hypoxia, perinatal asphyxia, and maternal pre-eclampsia), which may lead to in serum PCT levels similar to those of infected neonates. A normal PCT (cut-off: 0.5 µg/L using the BRAHMS PCTTM test) provides a strong negative predictive value for sepsis: procalcitonin-guided decision-making resulted in being superior to standard care in reducing antibiotic therapy in neonates born after 34 weeks of gestational age with suspected EOS in a multicenter randomized controlled trial (NeoPIns) [72].

Presepsin (P-SEP) is a soluble component of the CD14 receptor (sCD14) that is expressed on monocyte and macrophage cell walls. It serves as a receptor for the complex lipopolysaccharides-lipopolysaccharide-binding proteins (LPSs-LBPs) found on the outer wall of Gram-negative bacteria. An intracellular signal cascade mediated by Toll-like receptor 4 (TLR4) is activated by the contact between the CD14 and bacteria, with an early release of P-SEP in circulation [67,75]. Indeed, P-SEP rises in response to bacterial infections in about 2 h, with a peak at 3 h, and an 8-h halftime quicker than CPR and PCT, depending on the severity of the illness [76]. Cut-off values, specificity, and sensitivity varied significantly between research in relation to the infection type (typically both EOS and LOS considered combined) and methods of analysis (kind of samples, plasma, or whole blood) [67].

### 9.2. Biomarkers in Late-Onset Sepsis

When starting antibiotic therapy in newborns with suspected LOS, NICE guidelines suggest regularly tracking baseline concentrations of CRP [30]. Indeed, serial CRP monitoring may assist in evaluating the response to therapy, allowing for the termination of antibiotic medication when CRP declines [74], although we have no evidence that we should wait for negative CRP results before discontinuing antibiotic therapy.

Two CRP values < 10 mg/L acquired 24 h after the beginning of symptoms suggest an improbable bacterial infection, supporting the suspension of antibiotic therapy [77,78].

Similarly, a procalcitonin value ≤ 2.4 ng/mL carries a low risk of missing nosocomial sepsis [79]. Therefore, when PCT values are within normal limits, symptoms rapidly improve, and there are no positive results from blood cultures, antibiotic therapy can be stopped without too many doubts. PCT can also be useful in stopping antibiotic therapy earlier than the “standard” durations [45].

Concerning presepsin, multiple reports have reported that blood P-SEP levels steadily decrease with antibiotic therapy for both EOS and LOS [80,81,82]. So far, no focused studies have been conducted on the function of P-SEP in weaning neonates off empirical antibiotics, suggesting the necessity of performing additional research.

## 10. New Advances in Treatment

### 10.1. Antibiotic Regimens in Early-Onset and Late-Onset Sepsis

For early-onset sepsis (EOS), the combination of Ampicillin and Gentamicin is considered the first-choice empirical therapy [9,83]. In recent years, the utility of this dual regimen has come into doubt, given the large proportion of ampicillin resistance found with *Escherichia coli* and mounting indications of aminoglycoside resistance. Pre-term infants delivered after extended pre-term rupture of membranes, pre-natal beta-lactam antibiotic therapy, and subsequent concern for intraamniotic infection after birth are among those at highest risk [13]. Local antibiotic resistance should guide any change in the most common approaches (Table 2).

Whereas a high homogeneity has been identified in the empirical treatment of EOS, there is a substantial variability in LOS treatment, according to a recent European survey [84].

Indeed, in LOS episodes, causing pathogens are different, as the site of infection can also be different (blood, brain, lung, urinary tract, or skin and soft tissue), tailoring the antibiotic treatment according to the local microbiological epidemiology of the patient and the neonatal unit (Table 2), without a lack of consensus in favor of any particular regimen [85].

When there is evidence of a specific pathogen, antibiotic treatment should be modulated with a targeted therapy (Table 3) [45]. Available studies report practice variations in LOS treatment, with potential areas of improvement concerning the still wide use of vancomycin and third-generation cephalosporins [85,86].

Considering the always higher incidence of infections with multidrug-resistant (MDR) organisms across the world, novel antibiotics can represent a further therapeutic alternative, in particular against MDR Gram-negative bacteria (ceftazidime/avibactam, ceftolozane/tazobactam, cefiderocol, meropenem/vaborbactam, imipenem/relebactam) and Gram-positive bacteria with resistance of concern (ceftaroline and dalbavancin) [87]. However, further studies are needed to address their effectiveness and safety in newborns.

### 10.2. Antibiotic Lock Therapy

Furthermore, bacteria and fungi may bind to the internal surface of the central venous catheter (CVC) and form a biofilm where they are protected against natural immunity [88]. When a catheter-related bloodstream infection is diagnosed, and the blood culture is still positive after 48 h of antibiotics, the removal of the central line is strongly recommended. If the neonate still requires a CVC, a new one should be inserted, avoiding the previous site of placement [89]. In any case, sometimes the placement of a new central line can be difficult in critically ill infants, and a local administration of high-concentration of antibiotics into the catheter lumen (Antibiotic Lock Therapy, ALT) can allow the central line to be saved, although evidence surrounding this procedure is still limited (Table 4) [90].

Recently, the use of a 2% taurolidine lock solution for treating and preventing catheter-related bloodstream infections in neonates has been translated from adults’ experience: preliminary reports highlighted that its use is safe and appears to be a promising tool [91]. However, further research with a multicenter randomized controlled trial is warranted to prove its absolute efficacy in the prevention of catheter-related bloodstream infections.

### 10.3. Blood Product Transfusions

#### 10.3.1. Intravenous Immunoglobulins

Immunoglobulin G (IgG) antibodies are the only maternal antibodies that significantly reach the fetus through the human placenta, especially from the 32nd week of gestational age, and the endogenous IgG synthesis begins after the first weeks of life. This is why pre-term neonates are more susceptible to infections, and the administration of exogenous IgG antibodies has been postulated in neonatal sepsis [92].

However, to date, polyclonal intravenous immunoglobulins (IVIG) as an additional treatment seem not to lower mortality in neonates with sepsis [93].

The studies on IgM-enriched IVIG on newborns and adults are very small, and the overall data are insufficient to establish a strong conclusion of experimental benefits without well-designed RCTs [93]. However, significantly lower short-term mortality has been reported in IgM-enriched, IVIG-treated infants, particularly among infants affected by Candida spp. (10% vs. 53%) [94]. A further RCT on the use of IgM-enriched IVIG found that pre-term neonates treated by standard antibiotic protocol without immunoglobulins had an increased risk of death (11.76%) [95].

Adjunctive treatment with monoclonal IVIGs is still experimental [93].

#### 10.3.2. Platelet Transfusions

Sepsis-related thrombocytopenia is observed in about 50% of septic neonates. The association of increased mean platelet volume and thrombocytopenia has been associated with mortality because bone marrow exhaustion may represent an almost pre-terminal event. Gram-negative bacteria, *Staphylococcus* spp., and fungal infections (*Candida* spp.) are commonly associated with low platelet levels. Therefore, many neonates and infants undergo adult-derived platelet transfusions during neonatal sepsis [96].

The multicenter RCT PlaNeT-2 (Platelets for Neonatal Transfusion—Study 2) compared clinical outcomes in pre-term neonates (<34 weeks’ gestation at birth) and randomized 660 infants to receive prophylactic platelet transfusions to maintain platelet counts at or above either 25 × 10^9^/L (low threshold) or 50 × 10^9^/L (high threshold). Most infants (62% vs. 64%) were receiving antibiotic treatment for sepsis at random. Among them, those randomly assigned to receive platelet transfusions at a high threshold had a significantly higher rate of death or major bleeding within 28 days after randomization than those who received platelet transfusions at a low threshold [97].

#### 10.3.3. Exchange Transfusions

The meta-analysis of 14 studies (three RCTs, 11 controlled observational studies) on exchange transfusion (ET) in septic neonates revealed a reduction in mortality and a significant increase in pooled immunological parameters (immunoglobulin, complement, neutrophil levels) compared to controls. The descriptive study of nine uncontrolled observational studies indicated thrombocytopenia as the most frequently reported consequence. Given the heterogeneity and significant risk of bias, this meta-analysis did not advocate ET for newborn sepsis. Exchange transfusions may be considered on an individual basis in III-level hospitals [98].

## 11. HeRO Monitoring, Hemodynamic Evaluation, Artificial Intelligence, and Future Research Perspectives

The heart rate pattern seems to open a window into the functioning of the autonomic nervous system in pre-term newborns. When it becomes abnormal, it is a sign of an underlying pathology. It was reported that sepsis is often associated with a reduction in heart rate variability and transient decelerations [99]. Characterizing these abnormal heart rate characteristics (HRC) through mathematical models has led to the development of an HRC index, which represents the fold increase in the risk of sepsis. The strong influence of gestational age on positive and negative predictive values adds complexity to the interpretation of HRC indexes [100].

A specific monitor (HeRO^®^ monitor, Medical Predictive Science Corporation, Charlottesville, VA, USA) analyzes this heart rate variability and transforms it into a score which, if greater than 2, expresses the probability that the newborn will experience clinical deterioration associated with sepsis or other pathological conditions in the three to five days following the appearance of the anomaly [101]. It has been shown that monitoring pre-term infants with HeRO^®^ reduces mortality by 22% [102]. The advantages of this system would be the possibility of continuous, non-invasive monitoring at the patient’s bedside, the possibility of using data from an electrocardiography monitor placed at the patient’s bedside, no additional invasive procedures are necessary, and monitoring can be carried out on non-critical or even wary patients. The increase in the index has been shown to be associated with the presence of late-onset sepsis and an increase in mortality. It also appears that such monitoring is superior and additional to clinical and laboratory biomarkers in the diagnosis of sepsis. However, there are still lights and shadows regarding the actual benefit of this monitoring and, in a review, Fairchild summarized the advantages and disadvantages of HeRO^®^ monitoring [103].

Furthermore, referring to a trial conducted in nine NICUs from 2004 to 2010, it is possible that the monitored newborns were subjected to greater clinical evaluations and a significantly greater number of blood culture days of antibiotics than controls were. The conclusion was, however, that the use of non-invasive bedside monitors that predict potentially catastrophic pathologies is the future and that the use of HeRO^®^ appears to reduce mortality from sepsis. Also, the cardiac variability index and the nSOFA score were recently compared to blood cultures in VLBW newborns to evaluate the predictability of late-onset sepsis and mortality associated with sepsis. The conclusion was that the cardiac variability index provides an early warning of impending sepsis, whereas nSOFA after blood culture provides a better prediction of mortality [104].

Ever more clinicians are using neonatologist-performed echocardiography (NPE) to assess hemodynamic changes in septic neonates, who are likely to suffer from both left ventricle and right ventricle systolic, and left ventricle diastolic dysfunction. New methods like Tissue Doppler Imaging (TDI) may be more sensitive than conventional echocardiography in detecting myocardial dysfunction and driving a targeted hemodynamic management in the case of septic shock [105].

Artificial intelligence (AI) algorithms are an emerging way to find patterns of hemodynamic dysfunction in neonatal sepsis [106]. In the case of EOS, maternal and perinatal risk factors, clinical signs, and biomarkers can be integrated to create a new prediction model that can increase diagnostic accuracy and avoid the start of unnecessary antibiotics [107]. Similarly, in LOS episodes, these models may extract data from numerous sources (continuous monitoring of vital parameters, blood gas analysis, NPE) and assist clinical decision-making [108,109]. However, these machine learning models are still very expensive and deserve further evaluation before introducing them into clinical practice.

## 12. Conclusions

Neonatal sepsis, both EOS and LOS, is a devastating neonatal disease that involves three million newborns in the world and causes approximately 15% of all deaths at neonatal age. Sepsis is the second cause of death among newborns. There are no shared and accurate definitions of this pathology for the newborn, and this creates many obstacles to the study and research on this common and devastating condition. The diagnostic challenges and uncertain epidemiology of the disease necessarily arise from a variable definition of the disease.

The criteria for paediatric sepsis are not accurate for term infants and have not been examined in pre-term infants. The nSOFA score seems to represent a new opportunity to detect the worsening of sepsis and to develop a shared definition.

The extreme susceptibility of newborns to serious sepsis and its life-threatening nature leads neonatologists to the often-irrational use of antibiotics, a use that needs to be rationalized by therapeutic protocols specific to each neonatal care context and by the correct use of biomarkers and clinical practice for timely suspension of antibiotic therapies. Collaboration with the microbiology team is crucial. Randomized studies report the safety and effectiveness of PCT guidance for antibiotic discontinuation decisions but not for guiding treatment initiation in critically ill newborns. Biomarkers should not be used alone but in addition to microbiological cultures and clinical evaluation over time, which alone may be able to considerably reduce the duration of the antibiotic. Polyclonal intravenous immunoglobulins as an additional treatment seem not to influence mortality in neonates with sepsis. The use of artificial intelligence can help create treatment algorithms and guide us towards personalized medicine.

## Figures and Tables

**Table 1:** Main diagnostic tools available in the literature. *FiO_2_: fraction of inspired oxygen; NA: not applicable; SpO_2_: oxygen saturation measured by pulse oximetry*.

Diagnostic Tools	Criteria	Range
**Tollner sepsis score**	- Skin coloration (0–4): normal, moderate change, considerable change; - Microcirculation (0–3): normal, impaired, considerably impaired; - Metabolic acidosis (0–2): normal, pH > 7.2, pH < 7.2; - Muscular hypotonic (0–2): no, hypotonic, floppy; - Bradycardias (0–1): no, yes; - Apneic spells (0–1): no, yes; - Respiratory distress (0–2): no, yes; - Liver enlargement (0–1): 0–2 cm, 2–4 cm, >4 cm; - Gastrointestinal symptoms (0–1): no, yes; - White blood cell count (0–2): normal, leukocytosis, leukocytopenia; - Shift to the left (0–3): no, moderate, considerable; - Trombocytopenia (0–2): no, yes.	0 (best)–24 (worst)
**Hematologic Scoring System**	- Total WBC count (0–1): ≤5000/µL or ≥25,000 at birth, ≥30,000 at 12–24 h, ≥21,000 day 2 onwards; - Total PMN count (1–2): increased/decreased, no mature PMN seen; - Immature PMN count (1) if increased; - Immature: Total PMN ratio (1) if increased or ≥0.3; - Degenerative changes in PMN (1): toxic granules/cytoplasmic vacuoles; - Platelet count (1): ≤150,000/µL.	0 (best)–7 (worst)
**International Pediatric Consensus Conference statement on sepsis and organ dysfunction in Pediatrics**	Criteria for sepsis diagnosis, but without an objective numeric evaluation.	
**NNF clinical practice guidelines**	Criteria for sepsis diagnosis, but without an objective numeric evaluation.	
**NEO–KISS Sepsis score**	Criteria for sepsis diagnosis, but without an objective numeric evaluation.	
**Neonatal sequential organ failure assessment (nSOFA) score**	- Respiratory score (0–8): Mechanical ventilation SpO_2_/FiO_2_ - Cardiovascular score (0–4): Inotropes; systemic steroids. - Hematologic score (0–3): Platelet count.	0 (best)–15 (worst)
**NeoSep Severity Score**	- Birth weight (1–2): 1–2 kg, <1 kg; - Time in hospital (1) if ≤ 10 days; - Gestational age (1) if < 37 weeks; - Congenital anomalies (2); - Maximum respiratory support (2–3), based on oxygen supplementation, non-invasive ventilation (CPAP, BiPAP, HFNC), or invasive ventilation; - Temperature (1–2): <35.5 °C, ≥38–<39 °C, ≥ 39 °C; - Abdominal distension (1) if present; - Lethargy (1) or no/reduced movements (2); - Feeding difficulties (1); - Evidence of shock (1).	0 (best)–16 (worst)

**Table 2:** Suggested antibiotic regimens in early-onset and late-onset sepsis.

	Common Pathogens	Suggested Empiric Antibiotic Therapy
**Early-onset sepsis**		
Term and late pre-term infants (GA ≥ 34 weeks)	*Group B Streptococcus* *Escherichia coli*	**Penicillin** *(i.e., Ampicillin)* ***+*** **Aminoglycoside** *(i.e., Netilmicin, Gentamicin, or Amikacin upon local antibiotic resistance patterns)*
Pre-term infants (GA < 34 weeks)	*Escherichia coli* *Group B Streptococcus*	
**Late-onset sepsis**		
Term and late pre-term infants (GA ≥ 34 weeks)	*Escherichia coli* *Group B Streptococcus* Additional pathogens related to intensive care (*Staphylococcus aureus*, *Coagulase-negative Staphylococci*, *Enterobacter* spp., *Klebsiella* spp., *Pseudomonas* spp.)	***For infants admitted from the community:*** **Penicillin** *(i.e., Ampicillin)* ***+*** **Aminoglycoside** *(i.e., Netilmicin, Gentamicin, or Amikacin upon local antibiotic susceptibility patterns)* **[Alternative: Penicillin +** **Expanded-spectrum cephalosporin** *(i.e., Cefotaxime, Ceftazidime, or Cefepime upon local antibiotic susceptibility patterns)***]** ***For infants hospitalized since birth:*** **Oxacillin** **or Vancomycin** *(if the neonate is MRSA-colonized and/or critically ill at presentation)* ***+*** **Aminoglycoside** *(typically Gentamicin, or Amikacin, upon local antibiotic susceptibility patterns)* **or Carbapenem** *(i.e., Meropenem, if there is concern for meningitis caused by a multidrug-resistant, gram-negative organism)*
Pre-term infants (GA < 34 weeks)	*Coagulase-negative Staphylococci,* *Staphylococcus aureus* *Escherichia coli* *Klebsiella* spp. *Enterococcus* spp. *Group B Streptococcus*	

**Table 3:** Suggested pathogen-specific antibiotic regimens.

Pathogen-Specific Therapy	
Group B *Streptococcus*	Penicillin G or Ampicillin
*Escherichia coli*	Ampicillin (if Ampicillin-sensitive) Expanded-spectrum cephalosporin (i.e., cefotaxime, ceftazidime, or cefepime)
Multidrug-resistant gram-negative bacilli (including ESBL-producing organisms)	Meropenem
*Listeria monocytogenes*	Ampicillin and Gentamicin
*Methicillin-sensitive Staphylococcus aureus (MSSA)*	Ampicillin or Oxacillin
*Methicillin-resistant Staphylococcus aureus (MRSA)*	Vancomycin or Teicoplanin
*Vancomicin resistant Enterococci*	Linezolid or Daptomycin
*Carbapenem-resistant Gram-negative organisms (CROs)*	Colistin

**Table 4:** Some suggested antibiotic lock therapy regimens from our clinical practice.

Antibiotic	Dosage
Amikacin	3 mg/mL in 0.9% saline
Meropenem	2 mg/mL in 0.9% saline
Micafungin	5 mg/L (+70% ethanol)
Vancomycin	3 mg/mL in 0.9% saline

## Data Availability

No new data were created, and reported data were derived from cited references.
